# Influence of serum inflammatory cytokines on cytochrome P450 drug metabolising activity during breast cancer chemotherapy: a patient feasibility study

**DOI:** 10.1038/s41598-021-85048-1

**Published:** 2021-03-11

**Authors:** Rebekah L. I. Crake, Matthew R. Strother, Elisabeth Phillips, Matthew P. Doogue, Mei Zhang, Chris M. A. Frampton, Bridget A. Robinson, Margaret J. Currie

**Affiliations:** 1 Mackenzie Cancer Research Group, Christchurch, Department of Pathology and Biomedical Science, University of Otago Christchurch, 2 Riccarton Avenue, PO Box 4345, Christchurch, 8011 New Zealand; 2 Canterbury Regional Cancer and Haematology Services, Canterbury District Health Board, Christchurch, New Zealand; 3 Department of Medicine, University of Otago Christchurch, Christchurch, New Zealand; 4 Canterbury Health Laboratories, Christchurch, New Zealand

**Keywords:** Breast cancer, Chemotherapy, Cancer therapeutic resistance

## Abstract

Individual response to chemotherapy in patients with breast cancer is variable. Obesity and exercise are associated with better and worse outcomes, respectively, and it is known that both impact the systemic cytokine milieu. Cytochrome P450 (CYP) enzymes are responsible for the metabolism of many chemotherapy agents, and CYP enzyme activity has been shown to be modified by inflammatory cytokines in vitro and in vivo*.* Cytokine-associated changes in CYP metabolism may alter chemotherapy exposure, potentially affecting treatment response and patient survival. Therefore, better understanding of these biological relationships is required. This exploratory single arm open label trial investigated changes in in vivo CYP activity in twelve women treated for stage II or III breast cancer, and demonstrated for the first time the feasibility and safety of utilising the Inje phenotyping cocktail to measure CYP activity in cancer patients receiving chemotherapy. Relative CYP activity varied between participants, particularly for CYP2C9 and CYP2D6, and changes in serum concentrations of the inflammatory cytokine monocyte chemoattractant protein 1 inversely correlated to CYP3A4 activity during chemotherapy. Future use of phenotyping cocktails in a clinical oncology setting may help guide drug dosing and improve chemotherapy outcomes.

*Clinical Trial Registration*: Trial was retrospectively registered to the Australia New Zealand Clinical Trial Registry (ANZCTR). ACTRN12620000832976, 21 Aug 2020, https://www.anzctr.org.au/ACTRN12620000832976.aspx.

## Introduction

Hepatic cytochrome P450 (CYP) drug metabolising enzymes are involved in metabolism of chemotherapy drugs used to treat breast cancer (BC), such as tamoxifen, cyclophosphamide, dexamethasone, doxorubicin and paclitaxel^1,2^. These agents have narrow therapeutic indices, so even minor changes in CYP expression or activity may have significant impact on drug exposure, potentially impacting efficacy or toxicity^2^. In BC patients, there is high variability in intra- and inter-individual response to chemotherapy and this remains unpredictable at the individual patient level. It has been suggested that differences in chemotherapy metabolism may play a role in regulating response to chemotherapy between patients and within an individual over time^1–3^.

Drug metabolising CYP enzymes can be phenotypically grouped into three distinct subpopulations, extensive metabolisers (EMs), poor metabolisers (PMs) or intermediate metabolizers (IMs), and in the case of CYP2C19 and CYP2D6, ultra-rapid drug metabolisers (UMs)^4–7^. However, large genotype–phenotype association studies are inconsistent and at times conflicting^8,9^, demonstrating that genetics is not the sole cause of intra- and inter-individual differences in CYP activity. In particular, CYP phenotypes are not stable over time, indicating environmental factors are affecting phenotype^4^. Some environmental influences on phenotype are well described- such as co-administered drugs that inhibit or induce CYP metabolism^8,10^. However, even after accounting for these known environmental determinants of CYP activity, there remains substantial unexplained variability^8^.

Additional factors known to influence the expression and metabolic activity of hepatic CYP enzymes include age, sex, environmental exposures, epigenetic events, use of concurrent medications, and comorbid inflammatory diseases^4^. To date, very little is known about the effects of inflammatory cytokines on CYP activity in cancer patients. Evidence for inflammation-induced repression of hepatic CYP expression has been well-established in vitro^11–14^. Moreover, altered CYP activity has been observed in inflammatory-associated disease states, including HIV and hepatitis C infection, liver disease and cancer^15–21^. In cancer patients, systemic inflammation is a hallmark of tumour progression^22^, and is associated with poorer prognosis and outcome in both early and late stage cancer patients, and poorer responses to chemotherapy^23,24^. Prior work has shown increases in circulating C-reactive protein (CRP) inflammatory cytokine, correlate to decreased CYP3A4 activity in advanced cancer patients^25^.

Similarly to cancer, obesity is associated with a pro-inflammatory state. Obese patients are known to have chronically elevated circulating pro-inflammatory cytokines and acute phase response proteins such as leptin, resistin, tumour necrosis factor alpha (TNF-α), interleukin-1 beta (IL-1β), interleukin-6 (IL-6), monocyte chemoattractant protein 1 (MCP-1), CRP, serum amyloid A (SAA), and alpha 1 acid glycoprotein (AGP)^26–30^. In BC patients, obesity is associated with more advanced disease at diagnosis, increased risk of recurrence and metastasis, and poorer overall survival^31–34^. Individual studies and investigations of pooled data across studies describe inverse correlation between higher body mass index (BMI = kg/m^2^) at diagnosis and pathological complete response rates in BC patients treated with neoadjuvant chemotherapies^31,35–38^. These findings suggest obesity and inflammation may share a common pathway to poorer response via alterations in chemotherapy metabolism.

Conversely, aerobic and resistance exercise implemented during adjuvant chemotherapy has displayed a trend toward improved BC disease free survival outcomes, particularly in overweight or obese BC patients^39^. Two systematic reviews have concluded there is biological relevance in the association between exercise and BC outcome, as physical activity can alter levels of circulating inflammatory cytokines and other cancer-related biomarkers^40,41^. A number of biological mechanisms have been proposed to explain the complex relationships between systemic inflammation and BC outcome, many of these center on direct interactions between inflammatory markers and breast tumour cells^42,43^. Far less research has explored the impact of systemic inflammation on CYP-mediated hepatic metabolism of clinically important BC chemotherapy agents.

Previous studies in healthy participants have shown that the Inje cocktail (caffeine, losartan, omeprazole, dextromethorphan, and midazolam) can be used to simultaneously assess in vivo activity of CYP enzymes (CYP1A2, CYP2C9, CYP2C19, CYP2D6 and CYP3A4) responsible for most CYP mediated drug metabolism^44–48^. The use of the Inje cocktail to assess the effects of drugs or diseases on CYP activity has not yet been carried out in cancer patients receiving chemotherapy.

Therefore, this prospective single arm open label study recruited women being treated with either adjuvant or neo-adjuvant chemotherapy for stage II or III BC, with the aim to determine whether the use of the Inje cocktail to measure changes in CYP activity over time is feasible and safe. Further, additional samples were taken to explore if there was association between CYP activity and the concentration of circulating inflammatory cytokines during treatment.

## Results

### Study participants, sample collection and data generation

This study recruited a total of twelve women with stage II or III breast cancer (BC) receiving standard of care doxorubicin-cyclophosphamide and paclitaxel (AC-Pac) neoadjuvant (n = 6) and adjuvant (n = 6) chemotherapy at Christchurch Hospital. Participants were of (self-identified) NZ European (n = 9), NZ Māori (n = 1), English (n = 1), or other (n = 1) ethnicities, were aged 40 to 68 years, had BMI ranging from 20.7 to 39.4, body fat percentage ranging from 20.5% to 51.7%, and muscle mass ranging from 45.9% to 75.5% (Supplementary Table 1). None of the participants had chemotherapy dose-capped based on body surface area (BSA) (Supplementary Table 2).

Due to clinical complications, one participant was only able to complete two of the twelve scheduled paclitaxel doses, and therefore, this participant did not have measures for body morphometry, serum inflammatory cytokines, or CYP activity following dose 6 of paclitaxel, and step count data was not recorded during paclitaxel dose 6. Technical complications associated with the FitBit One devices prevented step count measures for three participants during AC cycle 1. Inflammatory cytokines could not be measured for one participant at baseline due to small serum volumes. Finally, owing to both clinical and/or technical complications during Inje cocktail phenotyping, two participants did not have serum or urine samples collected, and one additional participant did not have urine collected, following dose 6 of paclitaxel.

Based on the samples collected, changes in the metabolising activity of CYP2C19 and CYP3A4 were measured in serum samples from nine participants, and changes in the metabolising activity of CYP2C9 and CYP2D6 were measured in urine samples from eight participants. Variable concentrations of caffeine and paraxanthine were detected in serum samples from nine participants prior to probe drug administration before chemotherapy (Supplementary Table 3); indicative of baseline caffeine contamination. It was therefore deemed unacceptable for CYP1A2 metabolising activity to be assessed. Lastly, changes in serum angiopoietin-2 (ANG2), B-cell activating factor (BAFF), CRP, growth differentiation factor 15 (GDF-15), interleukin 10 (IL-10), and MCP-1 cytokine concentrations were measured in a total of ten participants, and changes in body morphometry were determined for a total of eleven participants.

### Intra-individual changes in CYP activity were observed during chemotherapy

**Figure 1:**
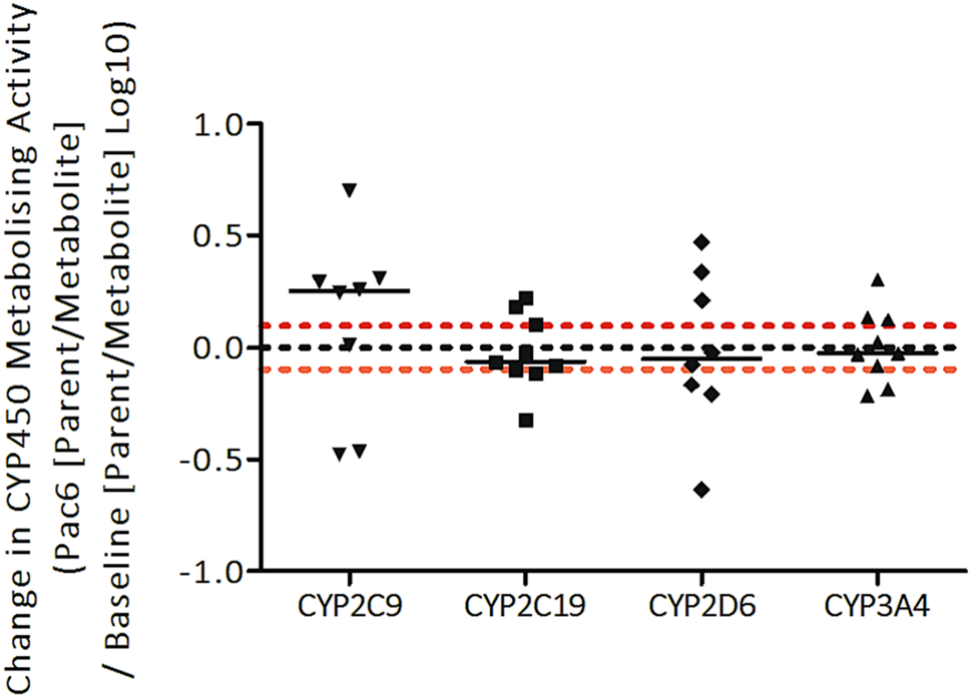
Changes in CYP metabolising ratios during chemotherapy for breast cancer. The change in CYP2C9 (n = 8), CYP2C19 (n = 9), CYP2D6 (n = 8) and CYP3A4 (n = 9) metabolising activity was determined by comparing the probe drug to metabolite ratios from after chemotherapy to before chemotherapy (log10). Horizontal black solid lines represent median values. Horizontal black dotted line represents no change in CYP metabolising activity from baseline to paclitaxel dose six, and points above or below the red dotted horizontal lines represent a decrease or increase, respectively, in CYP metabolising activity of 1.25-fold or greater (log10 of 0.80–1.25 =  ± 0.097) from before chemotherapy to after chemotherapy. Statistical analysis was performed using Wilcoxon matched-pairs signed rank testing, and significance was determined as *p* < 0.05.

In vivo phenotypic activity changes of paclitaxel dose 6 compared to baseline, is presented in Fig. 1. Time-related intra-patient variation in CYP activity, absolute value greater than 1.25-fold, was evident in seven participants for CYP2C9, five participants for CYP3A4, and six participants for CYP2C19 and CYP2D6 (Fig. 1). CYP2C9 and CYP2D6 had the largest variation in metabolising activity changes, with changes in metabolising ratios ranging from 0.33 to 5.02 for CYP2C9, and 0.23 to 2.94 for CYP2D6. Of the seven participants showing clinically meaningful alterations in CYP2C9 activity, two exhibited increased activity and five exhibited decreased activity (Fig. 1). Of the five participants that had clinically meaningful alterations in CYP3A4 activity, two had increased activity and three had decreased activity (Fig. 1). Finally, for the six participants that showed clinically meaningful changes in CYP2C19 and CYP2D6 activity throughout chemotherapy, three had increased activity and three had decreased activity (Fig. 1). However, at the population level, significant changes in CYP2C9, CYP2C19, CYP2D6 or CYP3A4 metabolising activity were not recorded during chemotherapy in this cohort of women with stage II or III BC (*p* > 0.05; Table 1). No adverse reactions to the Inje cocktail were recorded for any of the study participants.

**Table 1:** Changes in CYP metabolic ratios during chemotherapy for breast cancer.

Enzyme	Phenotyping	n	Before chemo phenotype ratio*	After chemo phenotype ratio*	After chemo/before chemo ratio (90% CI)	p**
CYP2C9	losartan/ E-3174	8	1.54	1.74	1.79 (0.79–2.78)	0.55
CYP2C19	omeprazole/5-hydroxyomeprazole	9	2.27	3.00	1.02 (0.78–1.26)	0.82
CYP2D6	dextromethorphan/dextrorphan	8	0.65	0.68	1.26 (0.64–1.87)	1.00
CYP3A4	midazolam/α-hydroxymidazolam	9	2.18	2.29	1.08 (0.81–1.35)	0.91

CI, confidence interval.

*Mean ratios.

**Statistical analysis was performed using Wilcoxon matched-pairs signed rank testing, and significance was determined as *p* < 0.05.

### Changes in serum cytokine concentrations were measured during chemotherapy

In participants with BMI > 30, eight of the 105 cytokines assessed using a cytokine array showed a 1.1-fold or higher increase in relative expression during chemotherapy, including B-cell activating factor (BAFF), growth/differentiation factor 15 (GDF-15), and angiopoietin-2 (ANG2) (Supplementary Figure 1). Based on these findings, and previous associations with inhibition of CYP expression and/or elevated adiposity^25,27,28,49^, the inflammatory cytokines ANG2, BAFF, CRP, GDF-15, IL-10, MCP-1, and TNF-α were selected for further analysis.

**Figure 2:**
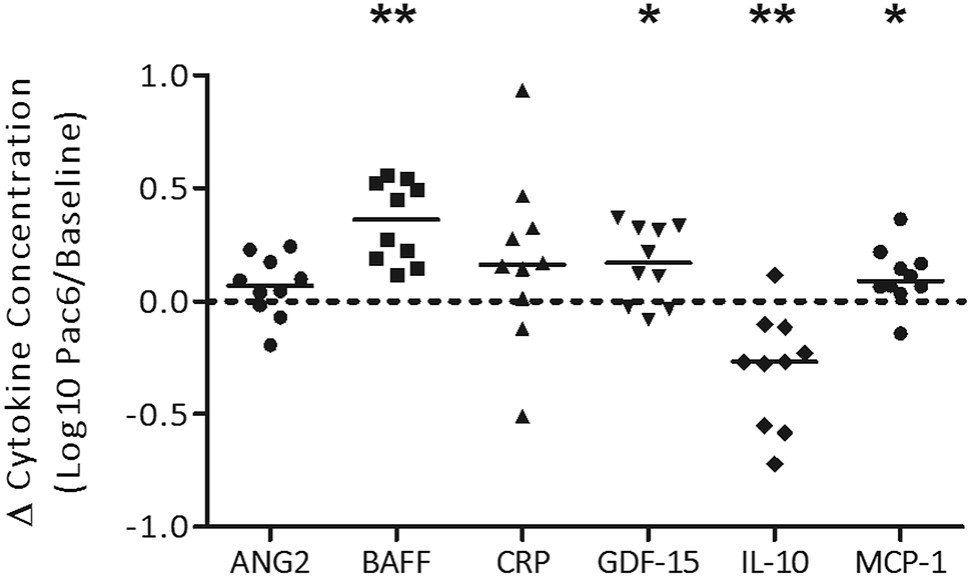
Change in circulating inflammatory cytokines measured during chemotherapy. Inflammatory cytokines ANG2, BAFF, CRP, GDF-15, IL-10, and MCP-1 were measured in participant serum using enzyme-linked immunosorbent assays before chemotherapy (n = 10), and after chemotherapy (after paclitaxel dose six; n = 10). Black horizontal solid lines represent median values. The black horizontal dotted line represents no difference in cytokine concentration from baseline to paclitaxel dose six, and points above or below the dotted line represent an increase or decrease in cytokine concentration during chemotherapy, respectively. Statistical analysis was performed using the Wilcoxon matched-pairs signed rank test. *p < 0.05; **p < 0.01.

Using ELISAs, serum cytokine concentrations between paclitaxel dose 6 and baseline showed a significant increase in the concentrations of BAFF, GDF-15, and MCP-1 (*p* < 0.05), and a significant decrease in IL-10 (*p* < 0.05; Fig. 2). Serum concentrations of ANG2 and CRP were unchanged during chemotherapy (Fig. 2), and TNF-α concentrations were below the assays detectable limits. Concentrations of inflammatory cytokines before chemotherapy (baseline) were not significantly different between participants treated with either neoadjuvant or adjuvant chemotherapy, suggesting surgery prior to starting chemotherapy did not influence the concentrations of circulating inflammatory cytokines (Supplementary Figure 2).

### Correlation between changes in CYP activity and serum cytokine concentrations

**Figure 3:**
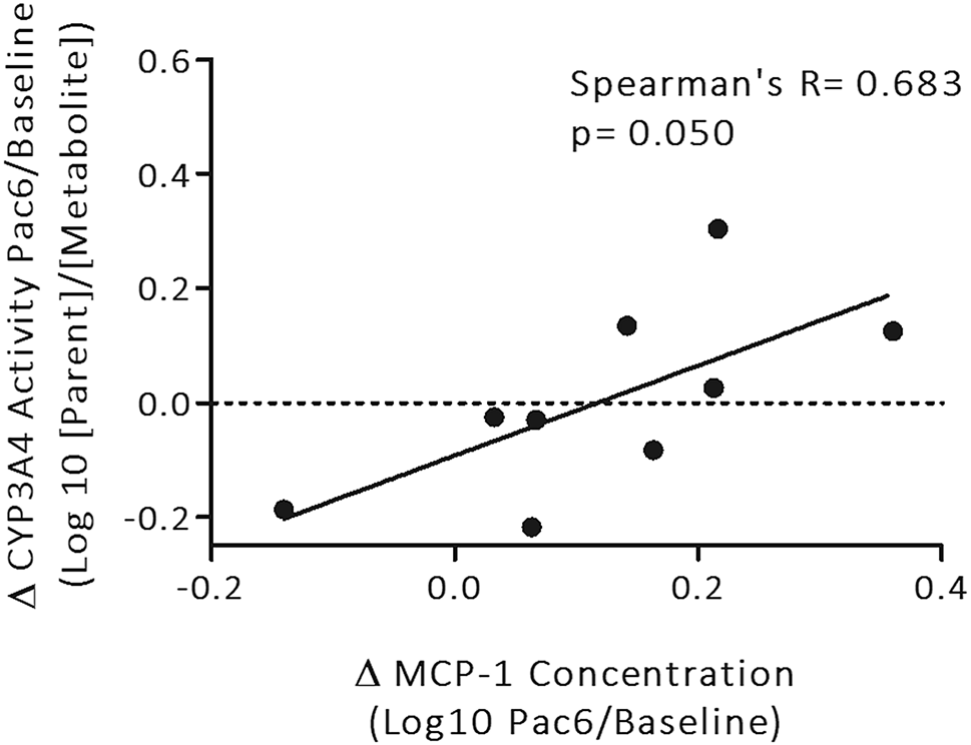
Correlation between changes in serum MCP-1 and changes in CYP3A4 metabolising activity during chemotherapy. The change in MCP-1 cytokine concentrations from before to after chemotherapy (log10), was correlated with the change in CYP3A4 metabolising activity from before to after chemotherapy (log10; n = 9). Black solid lines represent linear regression line of best fit. Horizontal black dotted lines represent no change in CYP3A4 metabolising activity from baseline to paclitaxel dose six, and points above or below this represent a decrease or increase in CYP3A4 metabolising activity, respectively. Statistical analysis was performed using Spearman correlation analysis, and significance was determined as *p* ≤ 0.05.

An increase in serum MCP-1 concentration was correlated with a decrease in CYP3A4 activity during chemotherapy (Spearman’s R = 0.683; Fig. 3). Decreases in CYP2C19 activity during chemotherapy trended towards a correlation with increasing serum concentrations of ANG2 (*p* = 0.121; Spearman’s R = 0.567), BAFF (*p* = 0.067; Spearman’s R = 0.650) and MCP-1 (*p* = 0.121; Spearman’s R = 0.567) (Supplementary Figure 3).

Changes in IL-10 concentrations were significantly positively correlated with changes in body fat percentage (Spearman’s R = 0.685, *p* = 0.025; Supplementary Figure 4). Changes in the remaining cytokines were not correlated with changes in body fat percentage.

Changes in inflammatory cytokines ANG2, BAFF, CRP, GDF-15, IL-10 and MCP-1 during chemotherapy were compared between participants that had either low or high daily step counts on average, throughout chemotherapy. Changes in serum cytokine concentrations during chemotherapy were not dependent on whether participants performed higher or lower levels of physical activity (Supplementary Figure 5).

An increase in BMI, but not body fat percentage, was observed during chemotherapy between baseline and paclitaxel dose 6 (Supplementary Figure 6A). Average daily step counts lowered between cycle 1 of AC and dose 1 of paclitaxel, and remained low until after paclitaxel dose 6 (Supplementary Figure 6B).

## Discussion

This study confirmed the safety and feasibility of using the Inje cocktail to assess the in vivo activity of CYP2C9, CYP2C19, CYP2D6, and CYP3A4 in women receiving chemotherapy for breast cancer (BC). Phenotyping cocktails are designed to limit the potential for interactions between the components, exhibit adequate specificity of agents to allow accurate CYP phenotyping, and minimize observable clinical effects^50^. The Inje cocktail developed by Ryu et al*.* concurrently assesses activity of CYP1A2, CYP2C9, CYP2C19, CYP2D6, and CYP3A4 using a single time point blood and urine sample collection following cocktail administration, with no notable probe-drug interactions or drug-associated adverse events observed^51^. Previous studies have used variations of the Inje cocktail to assess CYP activity in healthy human subjects^44–48^. However, the current study is the first to use this tool to assess in vivo activity of CYP1A2, CYP2C9, CYP2C19, CYP2D6, and CYP3A4 in a BC population while receiving chemotherapy.

In this study, the metabolising activity of CYP2C9, CYP2C19, CYP2D6, and CYP3A4 did not exhibit consistent population level changes during chemotherapy. Clinically important changes in activity, as defined by FDA, of CYP2C9, CYP2C19, CYP2D6 and CYP3A4 were observed in individual participants. For example, CYP2C9 activity, assessed using the metabolic ratio of losartan to E-3174 in urine, was altered by 1.25-fold or more throughout chemotherapy in seven out of the eight participants investigated, however, five of these participants exhibited decreased activity and the other two showed increased activity. These observations emphasise high variability in CYP activity over the course of chemotherapy treatment. Further studies may be able to better define patterns over time through more extensive sampling.

By concurrently assessing changes in the activity of CYP metabolising enzymes and concentrations of circulating inflammatory cytokines, this feasibility study was able to identify for the first time an inverse correlation between CYP3A4 activity and MCP-1 concentration during chemotherapy. Changes in the concentrations of the other circulating inflammatory cytokines were not correlated with alterations in CYP activity observed in this feasibility study. Prior to the current investigation, only one other study has documented an association between in vivo function of CYP3A4 and circulating inflammatory cytokines, in which patients with advanced cancer that had elevated systemic CRP also exhibited decreased CYP3A4 activity; as measured by the erythromycin breath test^25^. CYP3A4 is responsible for the metabolism of over 50% of the most widely administered BC therapeutic agents including cyclophosphamide, docetaxel, doxorubicin and paclitaxel; most of which are utilised in New Zealand clinics^52^. Therefore, unidentified decreases in CYP3A4 activity have the potential to alter chemotherapy metabolism in a manner that could unpredictably influence pathological responses and clinical outcomes for patients with BC.

MCP-1, also known as CCL2, is a C–C chemokine family member and a potent chemoattractant, regulating the recruitment and infiltration of monocytes to sites of inflammation^53^. MCP-1 has been implicated for its roles in a number of inflammatory related human diseases, predominantly HIV, cardiovascular disease, cancer, and obesity^53,54^. During obesity, MCP-1 concentrations are increased in inflamed adipose tissue and it has been observed that MCP-1 interacts with hypertrophic adipocytes and adipose tissue resident macrophages to upregulate production of itself and other inflammatory cytokines, such as TNF-α and IL-6; contributing to the low-grade systemic inflammatory condition characteristic of excessive adiposity^28,55,56^.

**Figure 4:**
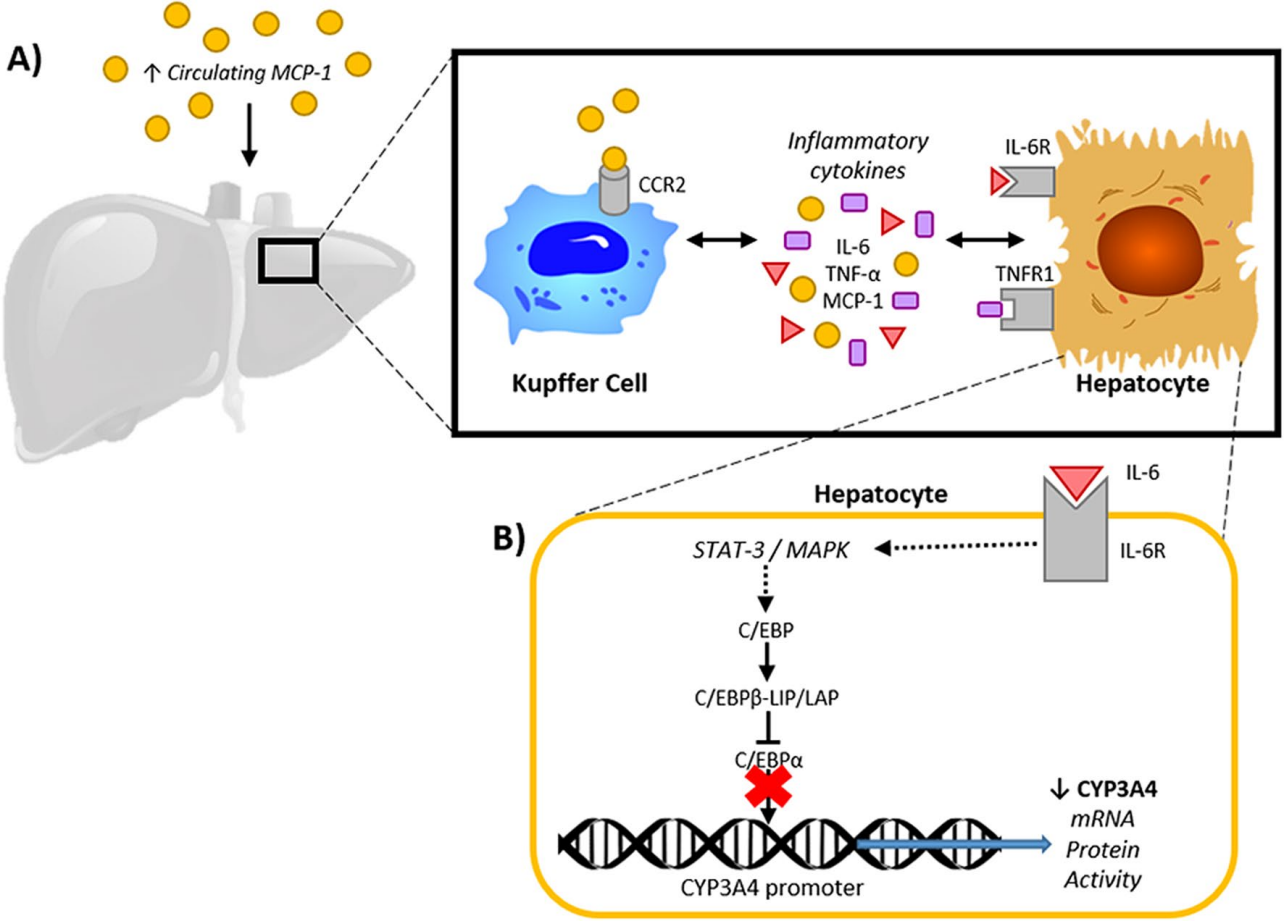
Monocyte chemoattractant protein 1 (MCP-1) mediated decrease in CYP3A4 activity in the liver of human breast cancer patients; proposed hypothesis. (**A**) The liver of breast cancer patients is exposed to increased circulating levels of MCP-1 during chemotherapy. Liver kupffer cells (hepatic macrophages) express the MCP-1 cell surface receptor C–C chemokine receptor type 2 (CCR2), and thus, MCP-1 can bind and induce an increase in the production of other inflammatory cytokines, such as interleukin 6 (IL-6) and tumour necrosis factor alpha (TNF-α), and further increase levels of MCP-1 molecules. IL-6 and TNF-α bind their membrane receptors, interleukin 6 receptor (IL-6R) and tumour necrosis factor receptor 1 (TNFR1), on the surface of nearby hepatocytes, inducing inflammatory signalling cascades that regulate CYP3A4 transcription. (**B**) Schematic of one of the mechanisms by which inflammatory cytokines inhibit CYP3A4 transcription; as reported by Jover et al.^58^, Intracellular signalling, following IL-6 binding, induces translation of CCAAT-enhancer-binding protein beta isoform LIP (C/EBPβ-LIP), an antagonist of CCAAT-enhancer-binding protein alpha (C/EBPα); C/EBPα is a known transcription factor that constitutively promotes CYP3A4 expression in hepatocytes^58^.

There are plausible biological mechanisms by which MCP-1 might influence the activity of CYP3A4 in hepatocytes. For example, MCP-1 might indirectly downregulate hepatocyte CYP3A4 activity by binding its cell surface receptor C–C chemokine receptor type 2 (CCR2) on Kupffer cell surfaces, increasing their production of inflammatory cytokines, such as IL-6 and TNF-α^57^. IL-6 can bind membrane receptors on nearby hepatocytes and promote signalling cascades previously documented to downregulate CYP3A4 transcription^58,59^ (Fig. 4). In order to test this hypothesis, the binding of MCP-1 to CCR2, and the subsequent release of inflammatory cytokines from liver Kupffer cells in BC patients receiving chemotherapy would need to be investigated.

It is possible that alterations in CYP expression and activity during chemotherapy may have been influenced by other inflammatory cytokines that were not measured in the current investigation. Previous in vitro investigations have identified suppression of CYP gene expression by IL-1β, IL-6, and TNF-α in human hepatocytes^11–14^. Findings from these in vitro studies showed that different cytokines influence CYP gene expression in a unique manner, whereby IL-6 is likely the predominant inflammatory cytokine involved in regulating CYP gene expression in humans^11–14^. In vivo studies assessing CYP activity in cancer patients have assessed concentrations of the pro-inflammatory mediators IL-1β, IL-6, IL-8, TNF-α, TGF, and the acute phase response protein, CRP^15,20,25^. The concentration of systemic inflammatory cytokines were not associated with differences in CYP2C19 and CYP2D6 activity^15,20^, but elevated CRP was shown to correlate with reduced CYP3A4 activity in patients with advanced cancer and was strongly positively correlated with levels of serum IL-6^25^. CRP is an acute phase response protein whose expression is triggered by increases in other circulating inflammatory cytokines, such as IL-6 and TNF-a^60,61^. The current study was not able to support previous findings, as serum CRP levels were not associated with alterations in CYP activity during chemotherapy. However, serum CRP was observed to increase during chemotherapy, suggesting a potential increase in the serum levels of other pro-inflammatory cytokines that were not measured in this investigation, that have the ability to suppress CYP expression and activity, such as IL-6. As the current study was the first to investigate changes in serum levels of BAFF, GDF-15 and MCP-1 in BC patients during chemotherapy, further research is required to validate and better understand the biological significance of these exploratory findings.

Similar to previous observations, participants in the current feasibility study exhibited an increase in BMI (Supplementary Figure 6A), a reduction in physical activity (Supplementary Figure 6B), increases in the circulating pro-inflammatory cytokines BAFF, GDF-15, and MCP-1 and a decrease in IL-10 during chemotherapy for BC (Fig. 2). Previous evidence suggests that physical activity with concurrent weight loss in women with or without BC, decreases levels of circulating pro-inflammatory cytokines and other biomarkers associated with obesity, such as IL-6, TNF-α, and leptin^62–65^. However, changes in circulating cytokine concentrations over chemotherapy were not found to be significantly associated with changes in body morphometry or differences in physical activity.

Although no known prototypical inducers or inhibitors of the studied CYP enzymes were being taken by participants in this study, there are still many other prescribed and ‘over the counter’ drugs that have the potential to induce and inhibit CYP enzymes in vivo^10^. Moreover, there are a number of cancer-related pathologies that are associated with increased levels of circulating inflammatory cytokines^66^. Therefore, in order to validate the findings from this feasibility study and assess the impact of other confounding factors on circulating inflammation and in vivo CYP activity, another larger and more comprehensive patient study is warranted. Despite being an exploratory study, the current study numbers align with the number of participants investigated in previous ‘Inje’ cocktail studies, which ranged from four to twenty-two participants^44–48,51^. However, the lack of statistical power associated with this study should be considered when interpreting the results of the statistical comparisons. Furthermore, this study did not report on participant survival outcomes, and whether recurrence and survival rates are affected by alterations in CYP activity in BC patients receiving chemotherapy, has yet to be directly assessed.

Overall, this study found that it is feasible to use a probe drug cocktail phenotyping approach to assess CYP activity changes in patients receiving chemotherapy for breast cancer. Inter-patient variability in CYP-mediated drug metabolism during chemotherapy treatment was evident, with individual patients showing clinically meaningful increases or decreases in activity for each CYP enzyme studied. Furthermore, this feasibility study observed for the first time that decreases in CYP3A4 activity over chemotherapy correlated with increases in serum levels of the inflammatory cytokine MCP-1; a relationship that requires validation in a larger cohort of patients. In the clinic, in vivo activity of CYP enzymes is most commonly predicted from an individual’s genotype, which underestimates the potential effects of a host of other factors, including inhibition of CYP expression and activity by increased levels of circulating inflammatory cytokines during chemotherapy for breast cancer. Results from this study support the use of phenotyping approaches in the clinical setting to better predict patient drug metabolism, enable more precise chemotherapy dosing and improve patient outcomes.

## Methods

### Study participants

Women with stage II or III breast cancer (BC), being treated with standard of care neoadjuvant or adjuvant doxorubicin-cyclophosphamide and paclitaxel (AC-Pac) chemotherapy^67^ at Christchurch Hospital, were recruited following informed written consent (New Zealand Health and Disability Ethics Committee (HDEC) approval: 16/CEN/116/AM01); all experiments were performed in accordance with the approved protocol. Participant inclusion criteria were adequate end organ function (creatinine levels ≤ 2 × upper limit of normal (ULN); haemoglobin > 90 g/L; systolic blood pressure > 90 mmHg; AST and ALT ≤ 3 × ULN; and bilirubin ≤ 2 × ULN), and patients were excluded who were taking medicines known to induce or inhibit CYP metabolism.

### Study design

Standard of care chemotherapy consisted of four 21-day cycles of AC, followed by administration of paclitaxel for twelve 7-day (weekly) cycles. Body morphometry measures (including body mass index (BMI) and body fat percentage using bioelectrical impedance analysis (BIA) on the Tanita Body Composition Analyser (Wedderburn, Hornby, Christchurch, NZ)), in vivo CYP phenotyping using the Inje cocktail (performed as described herein below), and two red-top 5 mL plain tubes (BD CAT coagulation) of blood were collected for inflammatory marker analysis prior to AC cycle 1 day 1 (baseline) and dose 6 day 7 of paclitaxel. The red-top tubes were processed to collect serum and frozen at -80 °C for subsequent inflammatory cytokine analysis. FitBit One devices were worn (unless specified in a device removal journal) following cycle 1 day 1 AC for 21 days, dose 1 day 1 of paclitaxel for 7 days, and dose 6 day 1 of paclitaxel for 7 days.

### Inje cocktail procedure

To simultaneously assess the in vivo function of five CYP liver enzymes, this study used a modified ‘Inje’ cocktail, adapted from Ryu et al. (2007)^51^. In brief, probe drugs were orally administered under direct observation in participants who had fasted from midnight on the day of cocktail administration. All participants were requested to refrain from consuming caffeine for 24 h prior to cocktail administration. Any concomitant medicines taken in the 24 h preceding the cocktail administration were recorded (Supplementary Table 4).

Tablets were taken with plain tap water, and liquids were taken and the administration cup was rinsed with 50 mL of plain water and re-taken. The cocktail components were: 100 mg caffeine tablet (Key Pharmaceuticals, Pty Ltd, Port Macquarie, NSW, Australia; Batch: P60064); 25 mg losartan tablet (Actavis, NJ, USA; Batch: GXM016002); 20 mg omeprazole tablet (Mylan, PA, USA; Batch: ZC16064B); 30 mg of dextromethorphan syrup (Pfizer, Sydney, NSW, Australia; Batch: 17RDX10A); and 1 mg of midazolam syrup (Claris Injectables Ltd, Ahmedabad, India; Batch: B5A0219). Participants were monitored by study nurses following probe drug cocktail administration, and any adverse reactions were recorded. Two red-top 5 mL plain tubes (BD CAT coagulation) of blood were taken at baseline (prior to administration of cocktail) and 4 h after the administration of the cocktail. Blood samples were processed by centrifugation at 1000×*g* for 15 min at 4 °C. Following centrifugation, serum was collected and stored at − 80 °C. 50 mL of urine was obtained before cocktail administration. Total urine was collected from 0 (cocktail administration) to 8 h, and following mixing, a 50 mL aliquot of this urine was taken and frozen at − 80 °C.

Measurements of the phenotyping cocktail drugs and metabolite concentrations in serum and urine were performed by the Department of Clinical Pharmacology (Department of Medicine, University of Otago Christchurch), using two in-house developed and validated LC-MS/MS assays^68^. Briefly, serum was used to assess the concentrations of caffeine, paraxanthine, omeprazole, 5-hydroxyomeprazole, midazolam and α-hydroxymidazolam, and urine was used for the measurement of losartan, E-3174, dextromethorphan and dextrorphan concentrations. Midazolam and α-hydroxymidazolam were analysed using the Agilent 6460 LC-MS/MS system, and dextromethorphan, dextrorphan, caffeine, paraxanthine, losartan, E-3174, omeprazole and 5-hydroxyomeprazole were analysed using the API 4000 LC-MS/MS system. The limits of the quantification in serum and urine were 0.2 ng/mL for midazolam and α-hydroxymidazolam, 1.25 ng/mL for dextromethorphan, and 5.0 ng/mL for caffeine, paraxanthine, losartan, E-3174, omeprazole, 5-hydroxyomeprazole, and dextrorphan. The intra- and inter-day coefficient of variation (CV%) over the analysed concentration ranges for all the compounds were < 10%.

Phenotypic activity of CYP2C9 and CYP2D6 were calculated using the log ratio of losartan/E-3174 and dextromethorphan/dextrorphan in urine, respectively. Phenotypic activity of CYP2C19 and CYP3A4 were calculated using the log ratio of omeprazole/5-hydroxyomeprazole and midazolam/α-hydroxymidazolam in serum, respectively. Changes in CYP enzyme activity during chemotherapy were assessed by measuring the difference between the metabolic ratios from after chemotherapy (following paclitaxel dose 6) to before chemotherapy (baseline). Phenotypic ratios greater than 1.25-fold for each individual participant, were categorised as clinically meaningful changes in CYP activity; as guided by the United States (US) Food and Drug Administration (FDA) in vivo drug metabolism and drug interaction study recommendations^69^.

### Serum inflammatory cytokine concentrations

The relative expression of 105 inflammatory cytokines was assessed using the Human XL Cytokine Array Kit (R&D Systems, Minneapolis, MN, USA), following manufacturer’s instructions, in pooled serum samples (4 × 25 µL = 100 µL total) from four participants that had a BMI greater than 30 (BMI > 30) at baseline and following paclitaxel dose 6.

Subsequently, serum cytokine concentrations were determined in all available patient serum samples collected at baseline and following paclitaxel dose 6, measuring human angiopoietin-2 (ANG2), B-cell activating factor (BAFF), C-reactive protein (CRP), growth differentiation factor 15 (GDF-15), interleukin 10 (IL-10), monocyte chemoattractant protein 1 (MCP-1), and tumour necrosis factor alpha (TNF-α), using the commercially available Quantikine ELISA kits (R&D Systems, Minneapolis, MN, USA), according to manufacturer’s instructions.

### Body morphometry measurements

Body morphometry measurements included height (cm), weight (kg), and fat and muscle composition (%). Fat and muscle composition was determined using bioelectrical impedance analysis (BIA). Intra-patient differences were calculated by comparing paclitaxel dose 6 to baseline measurements.

### Physical activity monitoring

Physical activity levels were determined from measures of daily step counts, using FitBit One devices. Each time the participant returned to clinic after wearing the FitBit One device, physical activity data, which included daily step count, was retrieved via* ‘*syncing’ of the device using FitBit Connect Desktop Client (RC Version 2.0.2.7066). Participants were grouped into low or high physical activity groups based on average daily step counts over chemotherapy, which were determined by combining the average daily step counts for AC cycle one, paclitaxel dose one, and paclitaxel dose six, and then splitting by median of the study participants (median average daily step count = 5537 steps).

### Statistical analysis

Data analysis was performed in GraphPad Prism Version 5.01. Given the relatively small sample size and the inherent variability associated with clinical data, all data analysis utilised non-parametric methods. Paired data were analysed using the Wilcoxon matched-pairs signed rank test. Mann Whitney U tests were used for the comparison of unpaired data. Associations between ordinal and continuous measures were evaluated using the Spearman’s correlation coefficient. For graphical purposes data are shown as log_10_ values and changes as differences in these log_10_ values. Statistical significance was considered as a *p* value < 0.05.

### Ethics approval and consent to participate

Ethical approval for this study was obtained through the New Zealand Health and Disability Ethics Committees (HDEC) Full Review Pathway (16/CEN/116/AM01), and participants were recruited following informed written consent. All methods were carried out in accordance with relevant guidelines and regulations.

### Consent for publication

The authors declare informed written consent was obtained from all study participants for use of de-identified clinicopathological data in this manuscript.

## Supplementary Information

Supplementary Information

## Data Availability

The authors declare that any data supporting the results reported in this article are not found elsewhere, and are only presented in this manuscript.
