# Supplementary material for: Influence of serum inflammatory cytokines on cytochrome P450 drug metabolising activity during breast cancer chemotherapy: a patient feasibility study
Source: Sci Rep. 2021 Mar 11;11:5648. doi: 10.1038/s41598-021-85048-1 (PMC7952716; doi:10.1038/s41598-021-85048-1)
Supplement: Supplementary file 1 — Supplementary Information [file 41598_2021_85048_MOESM1_ESM.pdf]

Supplementary information

Title: Influence of serum inflammatory cytokines on cytochrome P450 drug metabolising activity during breast cancer chemotherapy: A patient feasibility study

Authors and affiliations: *Rebekah L I Crake[1], Matthew R Strother[1,2,3], Elisabeth Phillips[1], Matthew P Doogue[3,4], Mei Zhang[3,4], Chris M A Frampton[3], Bridget A Robinson[1,2,3], Margaret J Currie[1].

[1]Mackenzie Cancer Research Group, Christchurch, Department of Pathology and Biomedical Science, University of Otago Christchurch, Christchurch, New Zealand
[2]Canterbury Regional Cancer and Haematology Services, Canterbury District Health Board, Christchurch, New Zealand
[3]Department of Medicine, University of Otago Christchurch, Christchurch, New Zealand
[4]Toxicology, Canterbury Health Laboratories, Christchurch, New Zealand

***Corresponding Author:** Rebekah Crake

rebekah.crake@otago.ac.nz
2 Riccarton Avenue
PO Box 4345
Christchurch 8011
New Zealand

Tel +64 3 364 0557

Supplementary Table 1. Patient information and baseline body morphometry measurements for the study participants.

Participant	Age	Ethnicity	Chemo Regime	Weight (kg)	Height (cm)	BMI	Body Fat %	Muscle Mass %
1	68	NZ European	Adjuvant	86.1	159	34.1	43.3	53.8
2	43	NZ European	Adjuvant	84.8	161	33.1	42.2	54.8
3	60	NZ European	Adjuvant	73	163	27.5	43.4	53.7
4	48	NZ Māori	Neoadjuvant	56.4	165	20.7	27.6	68.6
5	49	NZ European	Adjuvant	68.4	160	26.6	37.9	58.9
6	44	Other	Neoadjuvant	94.7	167	34.0	43.1	54.1
7	40	NZ European	Neoadjuvant	112.4	169	39.4	51.7	45.9
8	67	NZ European	Adjuvant	64.7	154	27.3	34.1	62.4
9	50	English	Adjuvant	61.2	166	22.2	28.7	67.6
10	50	NZ European	Neoadjuvant	72.2	163	27.2	20.5	75.5
11	42	NZ European	Neoadjuvant	61.3	155	25.5	33.5	63.1
12	65	NZ European	Neoadjuvant	73	154	30.8	41.9	55.1

BMI: Body mass index; **kg**: kilogram; **cm**: centimetre

Supplementary Table 2. Predicted doses based on BSA, and actual doses of adriamycin-cyclophosphamide and paclitaxel received by study participants.

Participant	BSA* (AC)	Predicted A (mg)	Actual A** (mg)	Predicted C (mg)	Actual C*** (mg)	BSA* (Pac)	Predicted Pac (mg)	Actual**** Pac (mg)
1	1.91	114	115	1140	1150	1.91	150	150
2	1.9	114	115	1140	1150	1.9	150	150
3	1.82	108	110	1080	1100	1.82	150	150
4	1.64	98.4	100	984	1000	1.66	132	130
5	1.74	104.4	105	1044	1050	1.84	145.6	140
6	2.07	124.2	120	1242	1200	2.1	168	170
7	2.2	132	135	1320	1350	2.2	176	180
8	1.64	98.4	95	984	950	1.63	130	130
9	1.72	103.2	100	1032	1000	1.71	136	140
10	1.79	107.4	105	1074	1050	1.79	143	140
11	1.6	96	95	960	950	1.62	130	130
12	1.73	103.8	105	1038	1050	1.7	136	140

BSA: body surface area; **AC:** adriamycin-cyclophosphamide; **Pac:** paclitaxel; **mg**: milligrams.
*BSA is calculated by multiplying height (cm) by weight (kg), dividing by 3600, and then calculating the square root.
**Adriamycin dosed based on BSA and rounded to the nearest 5mg
***Cyclophosphamide dosed based on BSA and rounded to the nearest 50mg
****Paclitaxel dosed based on BSA and rounded to the nearest 10mg

Supplementary Table 3. Concentrations of caffeine and paraxanthine in serum samples taken from each participant before probe drug administration.

	Before Chemotherapy (ng/mL)		After Chemotherapy (ng/mL)	
Participant	0Hr Caffeine	0Hr Paraxanthine	0Hr Caffeine	0Hr Paraxanthine
1	1876.7	1066.7	791.85	610.5
2	32.8	122.7	2066.7	1546.7
3	92.6	261.0	1846.7	926.7
4	< 5.0	91.5	< 5.0	< 5.0
5	70.8	131	< 5.0	15.2
6	< 5.0	< 5.0	< 5.0	55.3
7	41.2	538	n/a	n/a
8	259.7	553.7	115.5	289
9	1706.7	2366.7	63.3	289.3
11	217.3	389	n/a	n/a
12	224.7	471.3	445.3	744

<5.0: concentration of caffeine or paraxanthine was below the detectable limits of the assay.

0Hr: blood samples were drawn from participants prior to receiving the probe drug cocktail, and serum was prepared for analysis.

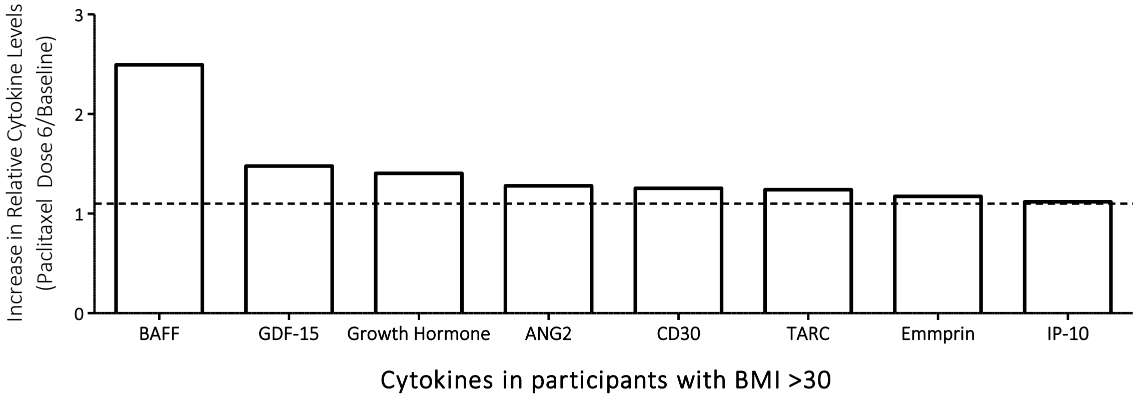

Supplementary Figure 1. Serum samples were assessed using a human cytokine array (105 cytokines) to measure increases from baseline (pooled sample from n= 4 participants) to paclitaxel dose six (pooled sample from n= 4 participants) in participants that have a BMI ≥ 30. Horizontal dotted lines represent a 1.1-fold change.

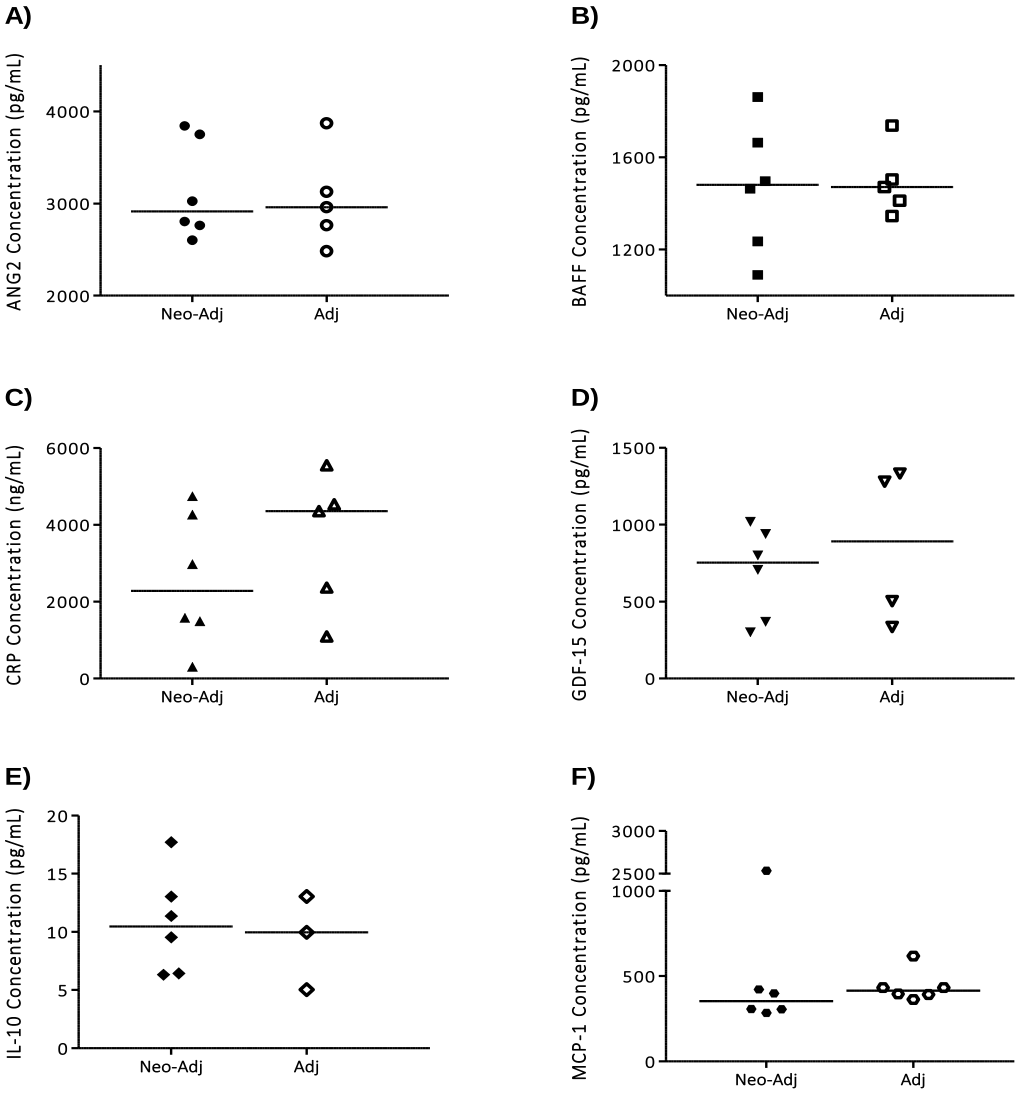

Supplementary Figure 2. Serum concentrations of circulating inflammatory cytokines (ANG2, BAFF, CRP, GDF-15, IL-10, and MCP-1) measured at baseline, were compared between participants treated with neoadjuvant (n=6) and adjuvant (n=6) chemotherapy. Black horizontal solid lines represent the median values. Statistical analysis was performed using Mann-Whitney U testing, and significance was determined as $p<0.05$.

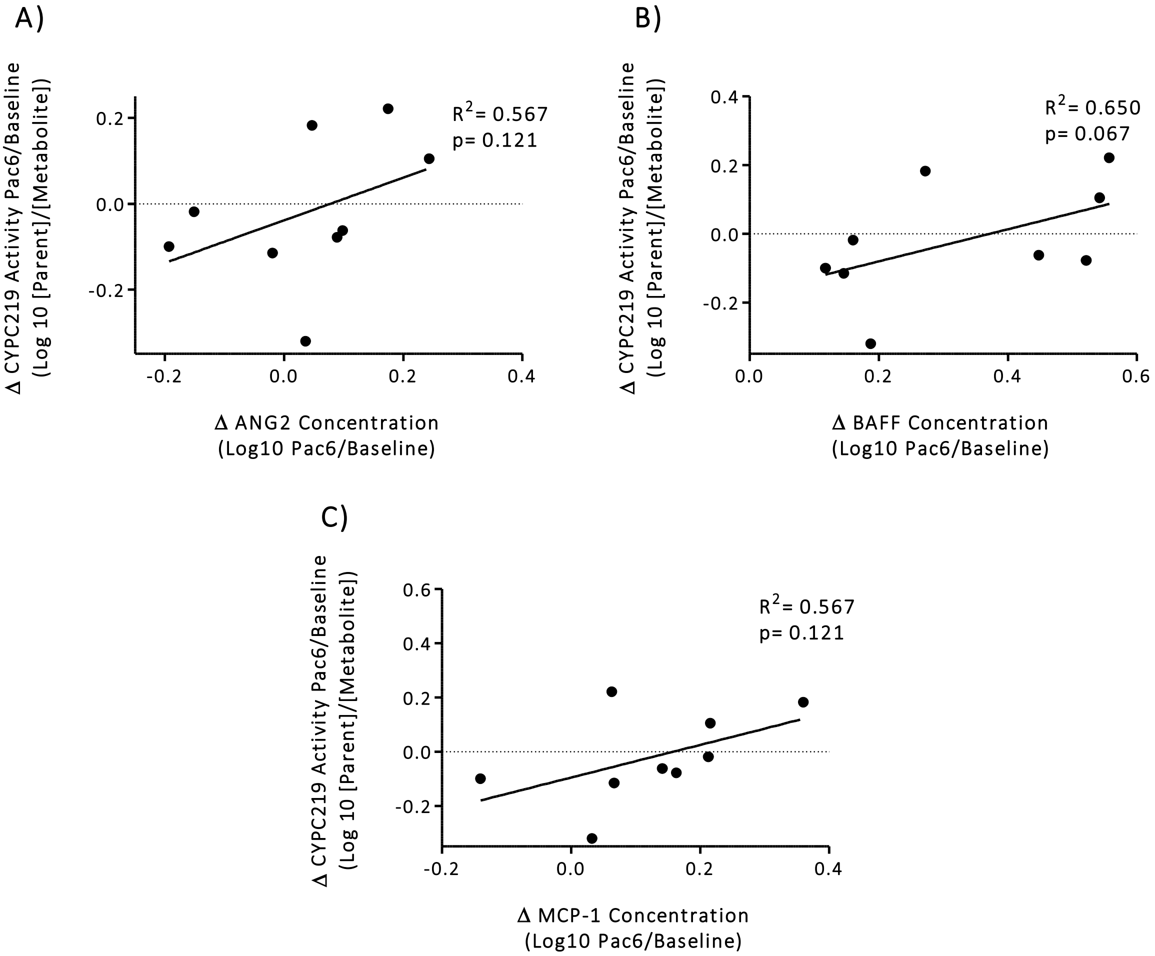

Supplementary Figure 3. Correlation between the change in serum ANG2, BAFF and MCP-1 and the change in CYP2C19 metabolising activity during chemotherapy. The change in A) ANG, B) BAFF, and C) MCP-1 cytokine concentrations from before to after chemotherapy (log10), was correlated with the change in CYP2C19 metabolising activity from before to after chemotherapy (log10; n= 9). Black solid lines represent linear regression line of best fit. Horizontal black dotted lines represent no change in CYP2C19 metabolising activity from baseline to paclitaxel dose six, and points above or below this represent a decrease or increase in CYP2C19 metabolising activity, respectively. Statistical analysis was performed using Spearman correlation analysis, and significance was determined as $p \leq 0.05$.

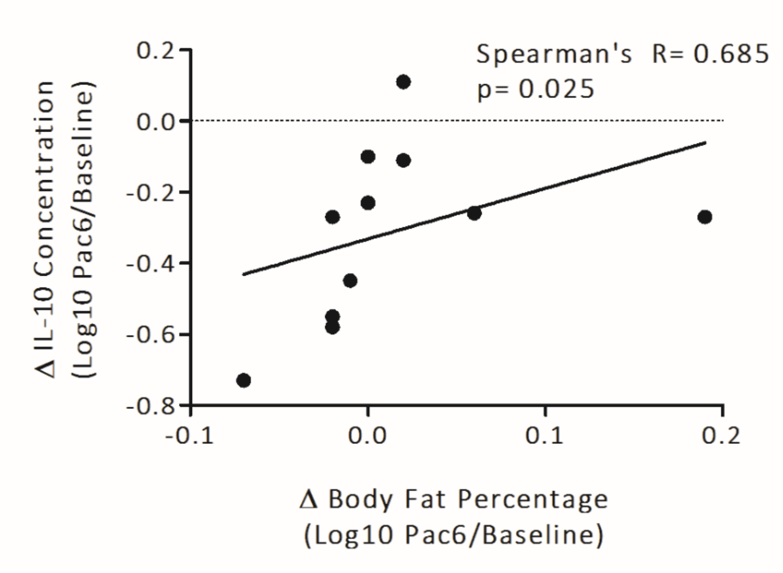

Supplementary Figure 4. Correlation between changes in serum IL-10 and changes in body fat percentage during chemotherapy. The change in serum IL-10 cytokine concentrations from before to after chemotherapy (log10), was correlated with the change in body fat percentage from before to after chemotherapy (log10; n= 11). Black solid lines represent linear regression line of best fit. Horizontal black dotted lines represent no change in inflammatory cytokine concentration from baseline to paclitaxel dose six, and points above or below this represent an increase or decrease in inflammatory cytokine concentration, respectively. Statistical analysis was performed using Spearman correlation analysis, and significance was determined as $p<0.05$.

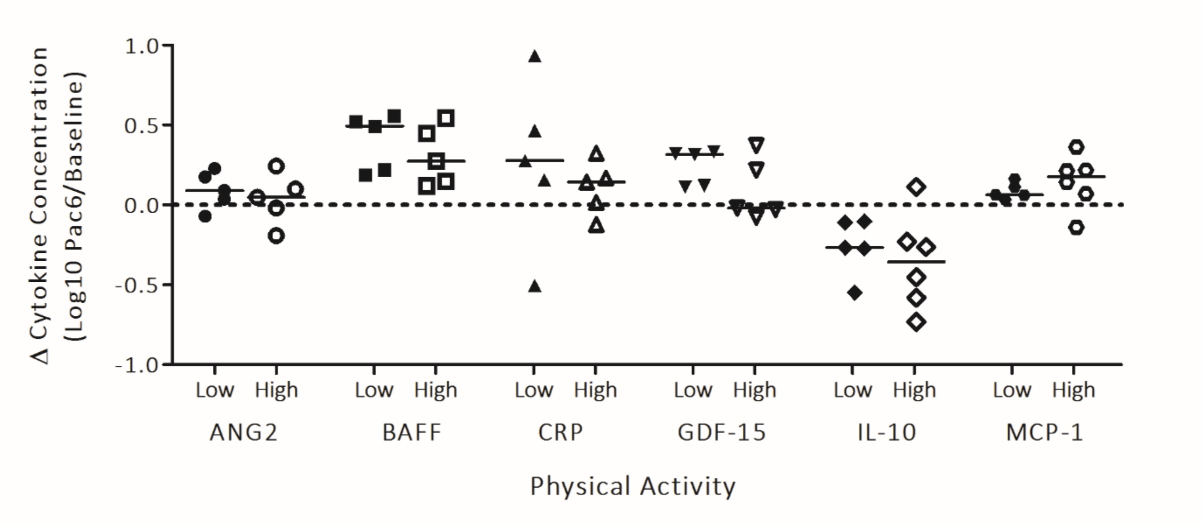

Supplementary Figure 5. Effect of physical activity levels on the change in circulating inflammatory cytokines measured during chemotherapy. The change in concentration of ANG2, BAFF, CRP, GDF-15, IL-10, and MCP-1 inflammatory cytokines from baseline to paclitaxel dose six, measured in serum using enzyme-linked immunosorbent assays, was compared between participants that had low (n= 5) or high (n= 5 for ANG2, BAFF, CRP and GDF-15, and n= 6 for IL-10 and MCP-1) average daily step counts (split by median= 5537 steps) recorded during AC cycle one, paclitaxel dose one and paclitaxel dose six using FitBit One devices. Black horizontal solid lines represent median values. The black horizontal dotted line represents no difference in cytokine concentration from baseline to paclitaxel dose six, and points above or below the dotted line represent an increase or decrease in cytokine concentration during chemotherapy, respectively. Statistical analysis was performed using Mann Whitney U testing, and significance was determined as $p<0.05$.

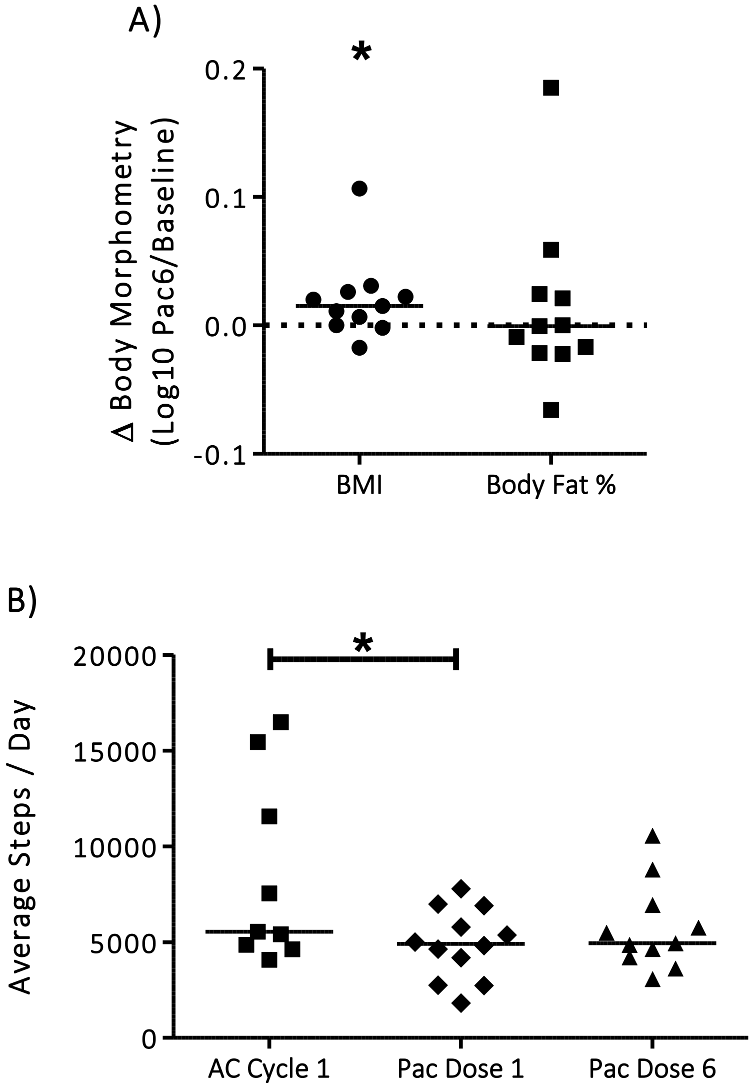

Supplementary Figure 6. Change in body morphometry (BMI and body fat percentage) and physical activity levels (average daily step counts) measured during chemotherapy. Changes in body morphometry were determined for eleven participants (n=11) from baseline to T1. Average daily step counts were determined for nine participants (n=9) over the 21 days of AC cycle 1, twelve participants (n=12) over the 7 days of paclitaxel dose 1, and eleven participants (n=11) over paclitaxel dose 7. Horizontal dotted line represents no change in body morphometry during chemotherapy. Horizontal solid line represents the median values. Statistical analysis was performed using Wilcoxon matched-pairs signed rank test. *p<0.05.

Supplementary Table 4. Concomitant medications taken in the 24 hours preceding cocktail administration, as recorded by study nurses according to participant recall.

Participant	Baseline	Paclitaxel Dose 6
1	none	none
2	Norty, omeprazole	none
3	plendel, bendrofluazide, paracetamol	none
4	Ibuprofen, Clonazepam	none
5	none	none
6	none	none
7	none	N/A
8	vit c (daily), green muscle extract (daily)	none
9	none	none
11	zopiclone or zopidone	N/A
12	atorvastatin	atorvastatin (40mg)
